# Active Spatial Perception in the Vibrissa Scanning Sensorimotor System

**DOI:** 10.1371/journal.pbio.0050015

**Published:** 2007-01-16

**Authors:** Samar B Mehta, Diane Whitmer, Rodolfo Figueroa, Ben A Williams, David Kleinfeld

**Affiliations:** 1 Neurosciences Graduate Program, University of California at San Diego, La Jolla, California, United States of America; 2 Computational Neurobiology Graduate Program, University of California at San Diego, La Jolla, California, United States of America; 3 Division of Biological Sciences, University of California at San Diego, La Jolla, California, United States of America; 4 Department of Physics, University of California at San Diego, La Jolla, California, United States of America; 5 Department of Psychology, University of California at San Diego, La Jolla, California, United States of America; 6 Center for Theoretical Biological Physics, University of California at San Diego, La Jolla, California, United States of America; Harvard University, United States of America

## Abstract

Haptic perception is an active process that provides an awareness of objects that are encountered as an organism scans its environment. In contrast to the sensation of touch produced by contact with an object, the perception of object location arises from the interpretation of tactile signals in the context of the changing configuration of the body. A discrete sensory representation and a low number of degrees of freedom in the motor plant make the ethologically prominent rat vibrissa system an ideal model for the study of the neuronal computations that underlie this perception. We found that rats with only a single vibrissa can combine touch and movement to distinguish the location of objects that vary in angle along the sweep of vibrissa motion. The patterns of this motion and of the corresponding behavioral responses show that rats can scan potential locations and decide which location contains a stimulus within 150 ms. This interval is consistent with just one to two whisk cycles and provides constraints on the underlying perceptual computation. Our data argue against strategies that do not require the integration of sensory and motor modalities. The ability to judge angular position with a single vibrissa thus connects previously described, motion-sensitive neurophysiological signals to perception in the behaving animal.

## Author Summary

Rats explore the world with their whiskers (vibrissae). Although the sensations of touch that an animal experiences while exploring an object either in front of its head or to its side can be similar, the two sensations tell the animal different things about its nearby environment. The translation from passive touch to knowledge of an object's location requires that the nervous system keep track of the location of the animal's body as it moves. We studied this process by restricting a rat's whisking information to that provided by a single actively moving vibrissa. We found that even with such limited information, rats can search for, locate, and differentiate objects near their heads with astonishing speed. Their behavior during this search reflects the computations performed by their nervous systems to locate objects based on touch, and this behavior demonstrates that rats keeps track of their vibrissa motion with a resolution of less than 0.1 s. Understanding how these computations are performed will bring us closer to understanding how the brain integrates the sense of touch with its sense of self.

## Abstract 3

Rats can localize objects with their specialized vibrissa system by the integration of feed-forward sensory events and motor feedback. This discovery provides a behavioral model for understanding how sensorimotor loops derive the perception of space from the sensation of touch.

## Introduction

“Neurophysiologists…have been reluctant to face up to [changes in anatomy from moment to moment] in explaining perception, for they know more about the anatomy of the eyes, ears, and skin than they do about the physiology of looking, listening, and touching” – J. J. Gibson [1].

The noted psychologist J. J. Gibson argued forty years ago [1] that the sensations produced by feed-forward transformation of inputs from neuronal exteroreceptors are distinct from the dynamic perception of the environment derived from the “neural loops of an active perceptual system”. Gibson's thesis was that the perception of the location of a contacted object, for example, is fundamentally different from the sense impressions that arise from skin mechanotransduction. Perception requires an integration of information across sensory and motor modalities that is not necessarily derived from successive transformations of the touch data alone. Studies in systems from posture control [2,3] to eye movement [4] have elucidated principles of such “neural loops” when used in motor systems with explicit sensory feedback. The work described here addresses the complementary case of positional context in a sensory system with explicit motor drive [5,6].

The rat vibrissa system, with its tactile hairs and their associated neuronal architecture, provides our prototype for this sensorimotor model. For nearly a century, researchers have compiled behavioral evidence that the vibrissae are both sensors and effectors in a complex sensory system that is able to identify and locate objects [7]. Although recent insights into the mechanical properties of the vibrissae [8,9] may explain the processing of qualities such as texture [10–13], few experiments, to our knowledge, have explicitly characterized the spatial information available from the vibrissae. Early work indicated that rats use this system for the detection of surfaces during navigation [14], and more recent studies have shown that the vibrissae provide information about object distance [15,16], shape [17], and orientation [18,19]. Few of these behaviors inherently engaged the sensorimotor nature of the system, and rats are known to perform some tasks, such as vibration [20] and bilateral distance [21] discrimination, with only passive vibrissa contacts. The system as a whole, in contrast, is fundamentally active. Of the many mammalian species with vibrissae [22], rats and related rodent species have specifically evolved the ability to sweep their vibrissae for dynamic exploration of the environment [23]. This ability leads us to question whether touch and motion are used in concert, in the spirit of Gibson, to form an “active perceptual system.”

Neurophysiologically, the sensory and motor processes are tightly interwoven. A nested series of loops, at levels from brainstem to cortex, connects the vibrissa sensory stream to a hierarchical motor drive that ordinarily produces a rhythmic rostrocaudal movement known as exploratory whisking [24]. Sensory inputs feed back onto motor areas at all levels and can alter motor output at even the lowest-order brainstem loops [25]. The information flow is bidirectional, however, and electrophysiological recordings have found neurons, again at levels from the brainstem to cortex, that encode the changing position of the vibrissae even in the absence of contact with an external object [26–29]. These cells complement somatosensory neurons that encode the qualities of contact and could provide the brain with an internal reference as self-generated motion modulates the external location that corresponds to contact.

**Figure 1:**
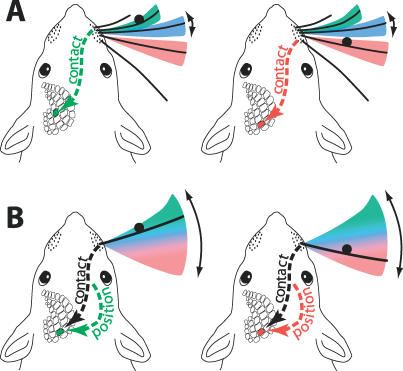
Two Localization Algorithms: Topographic Labeled-Line and Haptic-Sensing Each cartoon depicts an animal contacting a small object (black circle) and an idealization of the resulting neural streams (dashed arrows) afferent to the vibrissa somatosensory cortex. (A)A labeled-line strategy. During small motion, the location of an object is encoded in the identity of the vibrissa that contacts it. For clarity, only one row of vibrissae is shown; additional rows do not directly aid localization. (B)A sensorimotor strategy. During large motion, contact on a given vibrissa leaves object position ambiguous. Information about the position of that vibrissa at the time of contact resolves the confound. This scheme does not require multiple vibrissa.

The existence of position-sensitive signals does not prove that they are actually used for spatial perception. What has been lacking in the study of the neural computations that underlie the fusion of these touch and motion signals is a vibrissa-mediated behavior that requires sensorimotor integration. One such task would ask if a rat can use its vibrissae to differentiate between objects that differ only in rostrocaudal angle, i.e., azimuthal angle or angle along the rostrocaudal sweep of the vibrissae. However, the nervous system could in principle ignore motor information and solve this problem using the topography of the vibrissa array. When the motion of the vibrissae is comparable to their separation, the rostrocaudal position of an object can be judged from the peripheral origin of the touch signal (Figure 1A), in a scheme known as labeled-line encoding [30]. In this representation, the topographic identity of the cortical region activated by contact corresponds to object location, because each region of space is only scanned by a single sensor. An animal could thus use its vibrissa array to discriminate object angle if it used large-amplitude, exploratory motion to detect an object and switched to smaller motion for localization.

In the more typical exploratory whisking motion [31–34], vibrissa sweeps overlap, and the angular location that corresponds to contact requires information beyond the identity of the contacting vibrissa (Figure 1B). In this case, the presence of position-sensitive neurons suggests that the position of the vibrissae at the time of contact could provide context to interpret the contact event [35,36]. This sensorimotor approach falls under the rubric of haptic perception, as it is based on the awareness of the position of “an object relative to the body and the body relative to an object” [1]. Other algorithms derived from the touch sensation, such as duration of contact [28], are possible if different object positions result in reproducible differences in the structure of the contact event [37]. These approaches share the common feature that they do not depend on vibrissa identity and, therefore, unlike the labeled-line scheme, could be performed with a single vibrissa. To differentiate between these possibilities for spatial encoding and relating known sensorimotor physiological signals to a sensorimotor behavior, we thus ask if behaving rats are able to discriminate between objects at different rostrocaudal angles when restricted to the use of a single vibrissa.

## Results

**Figure 2:**
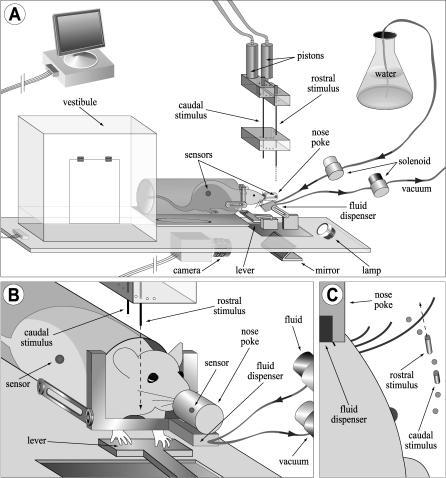
Apparatus for Behavioral Testing and Training (A) View of training arena. Animals were placed in the vestibule at the start of a session, and their position was monitored through infrared sensors. The U-shaped restraint bar blocked the tunnel while allowing access to the operant lever and nose poke. Discrimination trials started when an animal interrupted the nose poke sensor, causing either the rostral or caudal stimulus pin to descend into the vibrissa field. The stimuli were translated by air-driven pistons and positioned through a circular guide fixed relative to the nose poke (supporting parts omitted for clarity; see Figure S4). Lever presses in response to the ***S***+ stimulus, either rostral or caudal for each animal, were rewarded with a drop of water in the fluid dispenser. Any remaining fluid was withdrawn by vacuum at the end of the trial. An infrared lamp provided backlit contrast of the head and vibrissa for the camera recording the ventral view shown in (C). The entire arena was enclosed in a darkened, sound-attenuated chamber (not shown). (B) Detail of stimulus area from (A). (C) View of stimulus area from (A), as seen by the camera. The nose poke allowed reproducible positioning of the stimuli in head-centered coordinates (see also Figures 6A, 6B, 6D, 6E, and S1). [Original artwork: Jenny Groisman]

We tested 14 rats in an angle-based spatial discrimination task by using an apparatus designed for semi-automated training (Figure 2). Briefly, each animal was assigned two stimulus positions whose rostrocaudal angles differed by 15 °. One of these positions was designated the rewarded, or ***S***+*,* stimulus, and the other position was designated unrewarded, or ***S***−*.* The animal performed a series of trials in which it maintained a fixed head position while presented with a thin rod at one of these two positions. Under a go/no-go paradigm with a fixed ratio reinforcement schedule [38], the animal was required to respond to the ***S***+ position with a block of *L* lever presses within *T* seconds after stimulus presentation to obtain a fluid reward (see Methods); we used *T* = 8 s. Lever presses in the ***S***− condition were recorded but had no consequences. An animal was considered to discriminate between the two positions when multiple consecutive sessions showed a statistically significant difference between ***S***+ and ***S***− trials in the latency to complete this block response. We trained animals on this task with their full complement of vibrissae and then tested them while removing vibrissae, first down to a single row and finally to a single vibrissa.

**Figure 3:**
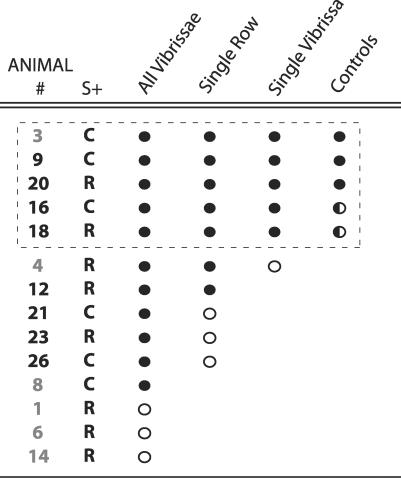
Summary of Performance Levels Achieved for All Animals Each row represents one of 14 rats tested on the spatial discrimination task. The first column gives an identifying number, and the second column gives the corresponding ***S***+ stimulus assignment (R for rostral and C for caudal). Animals with gray numbers were tested with response requirement *L* (Figure 7D) set to 1, and the remaining animals were tested with *L* = 4–6. The next three columns summarize performance as the number of intact vibrissae decreased. Filled circles indicate stable performance at a given level, and open circles imply that an animal was tested but did not achieve stable performance. “Stable performance” is defined here as statistically significant differences in ***S***+ and ***S−*** responses over multiple sessions. In two cases, rat number 8 and rat number 12, external circumstances caused the end of testing despite success at all attempted stages. The final column describes testing under various control conditions, and filled circles here indicate that a given animal passed all controls. The dashed box highlights those animals that succeeded in the task when limited to a single vibrissa. Among these rats, those that habitually sampled the stimuli with their head are shown with half-filled circles in the control column, since it was unclear whether this movement was involved in forming a spatial percept.

We considered it likely that a typical rat has the perceptual ability to perform this single vibrissa discrimination, despite the fact that only five of our initial 14 animals showed consistent behavioral differences between ***S***+ and ***S***− trials when tested with a single vibrissa (Figure 3). This attrition was largely due to a weakness in the behavioral measure used early in the study. The majority of the animals who did not progress to the single vibrissa condition were tested with a minimal lever press response requirement, *L* = 1 (Figure 3; animals outside the dashed box did not succeed in the single vibrissa task and animals with numbers in gray were tested with *L* = 1). The lack of demonstrated discrimination for these animals was likely because the low response requirement did not discourage animals from responding when they were able to predict that no reward would be forthcoming [38]. We thus raised the response requirement on *L* for later animals until differences in ***S***+ and ***S***− responses were seen (*L* = 4–6), and we then reliably measured discrimination for testing with all vibrissa intact (Figure 3; animals with numbers in black). This large increase in training success rate suggests that the measurements were limited by our ability to motivate the desired behavior rather than the animals' perceptual abilities.

### Time Scale of Single Vibrissa Discrimination

**Figure 4:**
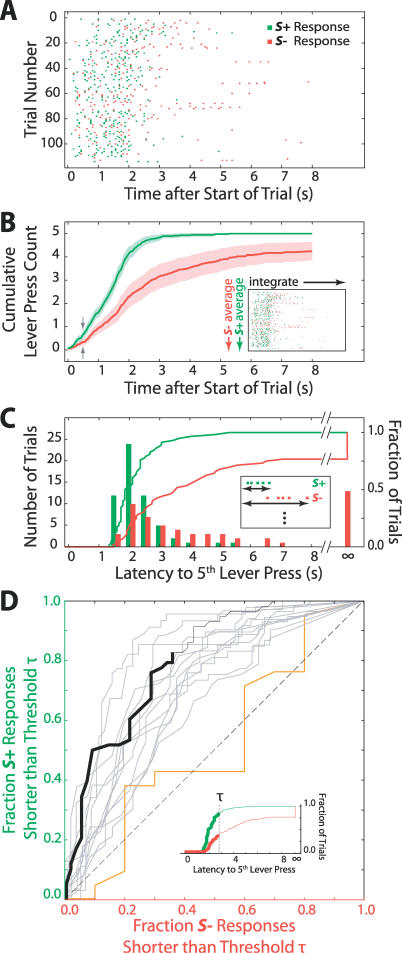
Temporal Profile of Behavioral Responses for One Session (A) Lever press responses in a session by rat number 20 restricted to the right C1 vibrissa. The trial length *T* was 8 s and response requirement *L* (Figure 7D) was five lever presses. Each row shows the first five lever presses in one trial, with responses from ***S***+ trials in green and those from ***S−*** trials in red. The fifth response in an ***S***+ trial was followed by a reward. This session consisted of 58 ***S***+ trials and 56 ***S−*** trials over 30 min. (B) Cumulative lever press counts from (A), averaged separately over ***S***+ and ***S−*** trials. The inset illustrates this data transformation. The green line and shaded region give the mean ± 2σ (standard error of mean) cumulative lever press counts for ***S***+ trials; equivalent data for ***S−*** trials are in red. The gray arrows at 0.5 s mark the time point after which the 2σ error regions remain nonoverlapping. (C) Distribution of latencies from the start of a trial to the fifth lever press, for the trials shown in (A). Trials with fewer than five responses are shown at infinite latency. The bars and left ticks show numbers of trials as a function of latency, and the lines and right ticks show the same data as cumulative distributions. The ***S***+ and ***S−*** distributions are statistically distinct (*p* < 0.001, two-sided K-S test). Green indicates ***S***+ and red indicates ***S−***. (D) Receiver operating characteristic curves summarizing differences between ***S***+ and ***S−*** latency distributions for multiple sessions. The fraction of ***S***+ trials with latencies below a threshold τ is plotted against the fraction of ***S−*** trials with latencies below the same τ; the curves are then constructed as τ varies parametrically. The result for the data from (C) is shown by the solid black line, where the heavy part of the line corresponds to the threshold τ having traversed the heavy parts of the lines in the inset data. The gray lines are from 12 subsequent single vibrissa sessions by the same animal (rat number 20). The orange line corresponds to the control session from Figure 5C. Identical ***S***+ and ***S−*** response distributions would yield the diagonal dashed line.

We compared the pattern of lever presses between ***S***+ and ***S−*** trials to estimate the time required to form a behavioral decision. Since the presence or absence of a reward after *L* lever presses could be used to distinguish the two stimulus conditions (Figure 4A), we considered only the first *L* responses in each trial in our analysis. The cumulative response count, averaged separately for ***S***+ and ***S−*** trials, quantified this time course for a given session (Figure 4B). ***S***+ trials resulted both in a higher response rate and in a lower latency to response onset relative to ***S−*** trials. In a typical session for the animal with the fastest responses (Rat number 20), the average response counts for the two conditions began to diverge approximately 250 ms after the start of a trial, and the 95% confidence regions became permanently nonoverlapping after 500 ms (Figure 4B; gray arrows). Thus, we could confidently measure a difference in the average response to ***S***+ and ***S−*** stimuli for this animal within 0.5 s of stimulus delivery.

The time in which these two response profiles diverge included the intervals needed both to form a sensory percept and to emit at least one lever press, i.e., *t*_diverge_ = *t*_perception_ + *t*_motor_. To place an upper bound on the interval *t*_perception_ needed for active sensing alone, we estimated the minimum time *t*_motor_ required for the motor act of pressing the lever from two observations. First, the slope of the linear part of the cumulative response for ***S***+ trials gave the mean response rate during sustained responding. This rate was roughly 2.5 lever presses per second, or 400 ms per average lever press (e.g., Figure 4B). Second, the shortest time taken by any animal to reach *L* = 5 lever presses was roughly 1.5 s (e.g., Figure 4C). Because few responses occurred in the first 250 ms of a trial (e.g., Figure 4A), this left 1.25 s for five lever presses, or 250 ms per lever press in the fastest cases. Both measures were based on blocks of lever presses that did not require further decision-making by the animal, and thus isolate the time *t*_motor_ to be 400 ms for typical responses and 250 ms for the fastest responses. Given *t*_diverge_ = 500 ms and using *t*_motor_ ≥ 250 ms, these data demonstrate that the complete interval *t*_perception_ required to detect the stimulus and form a percept of stimulus position is less than 250 ms.

### Variability across Sessions and Animals

**Figure 5:**
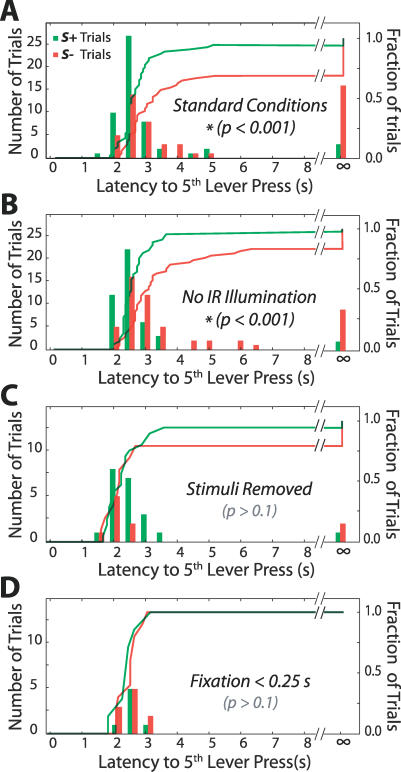
Controls for Extravibrissal Cues (A) Latency distributions for a standard session, performed by rat number 20 and similar to Figure 4C. This session contained 54 ***S***+ trials and 51 ***S−*** trials and showed a significant difference in the latency to the completion of a five–lever press response requirement when comparing responses from ***S***+ and ***S−*** trials (*p* < 0.001, two-sided K-S test). (B) A session performed without infrared (IR) illumination. This session contained 45 ***S***+ trials and 54 ***S−*** trials and showed a significant difference between ***S***+ and ***S−*** response latencies (*p* < 0.001, two-sided K-S test). (C) A session in which the stimulus pins were absent. This session contained 21 ***S***+ trials and ten ***S−*** trials and showed no significant difference between ***S***+ and ***S−*** response latencies (*p* > 0.1, two-sided K-S test). The number of trials was smaller here because the animal performed trials at a lower rate in this condition, and because the session was short (15 min versus 30 min above) to prevent extinction of the discrimination behavior. (D) Data from the session shown in (A) above, restricted to trials in which the animal broke fixation before the stimulus had fully extended. This condition included seven ***S***+ trials and ten ***S−*** trials and showed no significant difference between ***S***+ and ***S−*** response latencies (*p* > 0.1, two-sided K-S test).

The latency to reach the total required number of responses *L* in each trial served as our primary measure of behavioral discrimination (Figures 4C and 5A). A given session was considered to demonstrate stimulus discrimination when the ***S***+ and ***S−*** latency distributions significantly differed, as measured by a two-sided Kolmogorov-Smirnov (K-S) test. Consistent with the lack of an overt penalty in ***S−*** trials, we observed many more false-positive (type I) errors—where an animal completed *L* lever presses with short latency during an ***S−*** trial—than false-negative (type II) errors (Figures 4C, 5A, and 5B). Since the K-S statistic does not provide information about the types of errors, we confirmed consistent performance over multiple sessions by plotting receiver operating characteristic curves [39] for the detection of the ***S***+ stimulus (Figure 4D). Each receiver operating characteristic curve shows the dependency between false positive trials, i.e., ***S−*** trials with latencies shorter than a threshold τ, and true positive trials, i.e., ***S***+ trials with latencies shorter than τ, as the latency threshold τ is varied. Loosely, a steep slope near the left side of the graph indicates few type I errors, whereas a shallow slope near the right side of the graph indicates few type II errors; the integrated distance to the diagonal is a measure of the overall discriminability of the two distributions. The numbers of type I and type II errors fluctuated between sessions, but all animals showed a tendency for false-positive errors.

Further, although the bimodal nature of the latencies (Figure 4C) was consistent for all five animals in the single vibrissa task, the time at which ***S***+ and ***S−*** responses diverged fluctuated across sessions and animals. Each of the three animals that passed all controls, as described below, performed sessions in which the average ***S***+ and ***S−*** responses diverged within 650 ms. The remaining two animals took longer, i.e., greater than 1,000 ms, due to the head-turning behavior that is described below. These response times are consistent with an estimate of 250 ms for time needed for spatial discrimination.

Finally, the experimenter-defined association of stimulus position with reward condition varied across these five animals. Although all single vibrissa trials were performed at an angular separation of 15 °, the animals were tested on different absolute stimulus positions. For example, rat number 20 was tested with vibrissa C1 for ***S***+ set to +15 ° and ***S−*** set to 0 °, and rat number 9 was tested with vibrissa C2 for ***S***+ = −7.5 ° and ***S−*** = +7.5 °. In total, two animals had rostral ***S***+ assignments, and three had caudal ***S***+ assignments. We did not find any gross differences in training time or performance correlated with these parameters, which suggests that the absolute position of the ***S***+ and ***S−*** stimuli did not play a role in the discrimination.

### Controls

For the five animals that succeeded in the single vibrissa task over multiple sessions, we carried out three controls to verify that discrimination derived solely from vibrissa-mediated tactile cues. We first tested for visual cues. Although behavioral testing occurred in a dark chamber, a high-intensity infrared lamp with a peak wavelength of 850 nm was used for videography. We considered it possible, though unlikely [40], that residual visible light from this lamp was available to the animals. We thus tested performance in sessions for which the lamp was turned off, as illustrated by the data of Figure 5B. None of the five tested animals lost the ability to perform the task in the absence of the infrared illumination.

We next tested for a contribution from head movements. Stimuli were delivered after an animal fixed its head position in the nose poke and were retracted when fixation was lost (Figure S2); but mechanical lags created an approximately 150-ms window after an animal left the nose poke during which the stimulus was not fully retracted. Video observation showed that two of the five animals that succeeded in discrimination with a single vibrissa habitually probed the stimuli with their snouts after making vibrissa contact. It was not clear whether this movement was needed to aid stimulus localization, or whether it occurred as a reflex motion after the location had already been judged. Since we could not rule out the former possibility, we considered these cases ambiguous (Figure 3; half-shaded circles).

We finally tested the remaining three animals for the use of auditory and vibrational cues. The pistons used to deliver the stimuli were damped to reduce vibrations, and testing was carried out in the presence of an audio mask to interfere with any sound differences in ***S***+ and ***S−*** stimulus delivery. To confirm that discrimination was not based on residual indirect cues arising from stimulus motion, each animal was tested in a session where the pistons functioned as usual but the stimulus pins were absent. A resulting degradation in performance indicated that tactile contact with the vibrissa was required (see Figure 5C and the orange line in Figure 4D). None of the three tested animals showed significant discrimination in these control sessions.

In several sessions, we were able to further control for vibration cues by restricting our attention to trials in which the animal broke fixation less than 250 ms after the start of a trial. In these cases, the stimulus was retracted before it fully entered the vibrissa field (Figure S2). This allowed the pistons to travel over nearly their entire range but left insufficient time for the vibrissae to encounter the stimuli. In sessions with sufficient numbers of these “jump-the-gun” trials, we verified that discrimination performance was at or near chance with this limited opportunity for vibrissa contact, as seen in Figure 5D.

### Whisking Strategies

**Figure 6:**
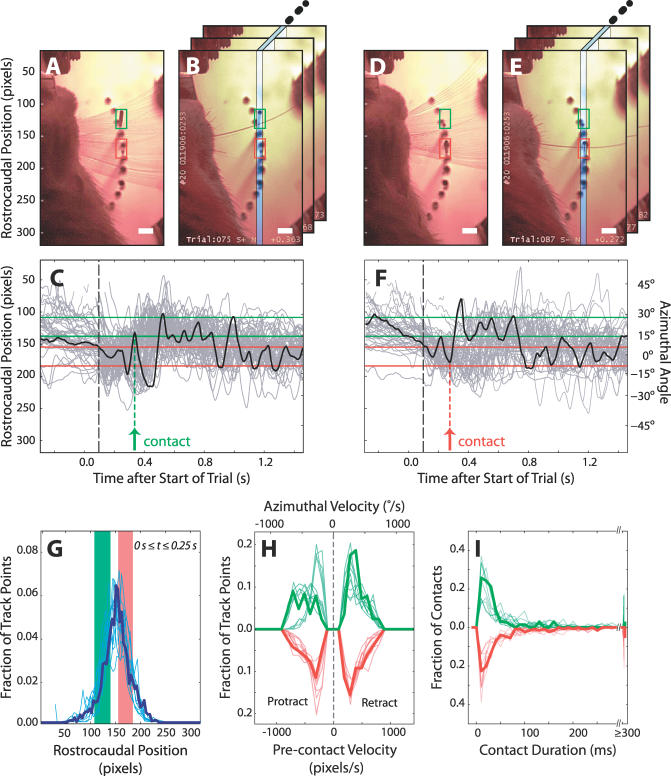
Patterns of Vibrissa Motion during Discrimination The data in (A) to (F) are from the session analyzed in Figure 4, with ***S***+ stimulus (rostral for rat number 20) at +15° and ***S−*** stimulus at 0°. The scale bar in the photographs is equal to 4 mm. (A) Projection of 400 frames from a single ***S***+ trial. This image shows the range of vibrissa positions in the interval from −0.5 to +1.5 s relative to the start of a trial; this range arises from whisking movements as well as small head motion and translations of the mystacial pad. The ***S***+ stimulus neighborhood is indicated by a green box, and the ***S−*** stimulus region is in red. The dark line in the green box is due to stimulus motion; compare to the green boxes in (B) and (E), which show fully extended and retracted positions, respectively. Stimulus displacement appears smaller than the true 5-cm travel because the motion was nearly normal to the focal plane (Figures 2A and 2C). (B) Single frame in which the vibrissa contacted the ***S***+ stimulus, taken from the trial in (A). The blue rectangle indicates the region in which vibrissa position was estimated for (C). (C) Vibrissa position as a function of time. The green and red bands correspond to the vertical extent of the similarly colored boxes in (A). The gray lines give position traces from 58 ***S***+ trials, and the black line highlights the trial shown in (A). The stimulus is not seen here, but both rostral and caudal stimuli started their descent at ∼30 ms and reached full extension at ∼300 ms (Figure S2). The stimuli were fully withdrawn within 150 ms of the end of nose poke fixation; this occurred at a median of 315 ms for ***S***+ trials and 530 ms for ***S−*** trials. The green arrow marks the ***S***+ contact in (B), and the dashed black line demonstrates the ∼100-ms delay in the onset of whisking after the start of a trial. Breaks in the lines are due to tracking errors. (D and E) Video images from an ***S−*** trial, analogous to (A) and (B). (F)Vibrissa position traces for 56 ***S−*** trials, analogous to (C). The red arrow marks the ***S−*** contact in (E). (G) Summary of all tracked vibrissa positions from 0–0.25 s after the start of each trial, including both ***S***+ and ***S−*** trials. The green and red bands correspond to the vertical extent of the corresponding regions in (A) and (C) and show that the vibrissa scanned both stimulus positions. The thick line is derived from the session analyzed in (A) through (F). The thin lines are taken from five more sessions by rat number 20 and four by rat number 9; these lines were scaled to align with the ***S***+ and ***S−*** bands drawn for the session represented by the thick line. (H) Precontact vibrissa velocities. Bold lines show the distribution of angular velocities as the vibrissa approached the stimulus for all contacts from (C) and (F), excluding intervals in which the vibrissae were in contact with a stimulus and thus not appreciably moving (defined as a velocity ≤1 pixel per 5 ms frame). The thin lines are taken from the sessions considered in (G). In each case, the ***S***+ and ***S−*** stimuli are associated both with contacts of positive velocity (i.e., retraction) and of negative velocity (i.e., protraction). Data from ***S***+ trials are in green and data from ***S−*** trials are in red. (I) Vibrissa contact event durations. Bold lines show the distribution of all contact durations from (C) and (F). The thin lines are taken from the sessions considered in (G). The traces vary in the width of the early peak and the fraction of sustained contacts, but none shows marked differences between ***S***+ and ***S−*** distributions. ***S***+ contacts are in green and ***S−*** contacts are in red.

To characterize the formation of the spatial percept in the interval *t*_perception_ preceding the motor response, we made high-speed infrared video recordings of vibrissa motion for two of the three animals that passed all of the controls, i.e., rat number 9 and rat number 20 (Figure 6A, 6B, 6D, and 6E). Vibrissae positions estimated from these recordings typically showed a slow drift until approximately 100 ms into the trial, followed by larger-amplitude, but often nonsinusoidal, vibrissa motion on a changing baseline (Figure 6C and 6F). This interval reflects the delay after the stimulus begins to descend before the animal realizes that a trial has begun. Thus, further subdividing *t*_perception_ = *t*_delay-to-start-of-search_ + *t*_active-sensing_ yields an estimate for the average time required to actively scan for a stimulus as *t*_active-sensing_ ≈ 150 ms.

Constraints on sensorimotor algorithms can be obtained from a more detailed analysis of patterns in the vibrissa motion. An animal could adopt a purely motor strategy by repeatedly positioning its vibrissa in the expected location of one of the stimuli and then simply detecting contact. To rule out this approach, we examined the distribution of all vibrissa positions in the first 250 ms of the trials, an interval chosen because it included few stimulus contacts. This distribution was, in all cases, centered between the two stimuli, indicating that vibrissa positions spanned both potential locations rather than habitually preferring one (Figure 6G). In a related strategy, an animal could center its vibrissa between the ***S***+ and ***S−*** stimulus locations and then associate contact during protraction with the more rostral stimulus and contact during retraction with the more caudal stimulus. As a test of this directional bias, we calculated the velocity of the vibrissa just before contact with ***S***+ and ***S−*** stimuli. Contact at each location occurs during both protracting and retracting movements, so that the direction of contact does not encode stimulus location (Figure 6H). This is consistent with the further observation that the distribution of vibrissa positions in the first 250 ms of the trials extends beyond the location of the two stimuli (Figure 6G), so that contacts can occur in both movement directions.

Another class of algorithms that does not require integration of positional and contact information depends on the duration of vibrissa contact [28,37,41]. For periodic vibrissa motion, the time between contact onset and offset could be transformed into object position. Such a scheme might be accomplished more generally for any vibrissa motion in which contact duration is a monotonic function of vibrissa angle. We thus asked if the distribution of contact event durations was different for ***S***+ and ***S−*** trials. These contacts were largely brief events of less than 50 ms, although extended contacts of greater than 300 ms occasionally occurred (Figure 6I). This overall distribution of contact durations is consistent with earlier measurements (Figure 3A from Sachdev et al. [42]) but the precise times varied broadly from trial to trial. Thus, for all animals, the variability of contact duration was large relative to any separation between the peaks of the distributions for the ***S***+ and ***S−*** conditions. In light of this variability, we consider an algorithm based on the duration of contact unlikely to account for the observed behavioral performance.

## Discussion

We have demonstrated that rats with a single vibrissa can respond to differences in the position of external objects that differ only in angle along the direction of vibrissa motion. A rat with a full array of vibrissa could in principle perform this task by limiting vibrissa motion to nonoverlapping fields and by using the collection of vibrissa columns, or arcs, as a spatial sensory array to test multiple locations in parallel (Figure 1A). The ability of animals to identify spatial angle when restricted to a single vibrissa argues that this sensorimotor system is able to determine the position of contact with an object while serially scanning a single sensor. The motion of the vibrissae as the animals performed this task was observed to span both stimulus locations prior to contact (Figure 6G), make contact during both protraction and retraction (Figure 6H), and remain in contact with both ***S***+ and ***S−*** stimuli for similar durations (Figure 6I). Although these experiments did not focus on discrimination using multiple vibrissae, we note that animals did not show any marked change in strategy or accuracy as they transitioned from single row to single vibrissa testing. This suggests that animals used the scanning strategy with multiple vibrissae as well, but the large variability in performance may have obscured indications of subtle changes in strategy.

The time scale for tactile search was estimated from the measured behavioral delays. Animals could selectively initiate a motor response in as little as 250 ms after the start of an ***S***+ trial (Figures 4B, 4C, and S3). In this short interval, we typically observed delays of 100 ms from the start of a trial before the vibrissa started a large amplitude scanning motion (Figure 6C and 6F). Taken together, these intervals imply that the time *t*_active-sensing_ including the entire sensorimotor process of motor scanning, object detection, and spatial categorization can be as short as 150 ms. Given a typical frequency of exploratory whisking in the range of 7–15 Hz [33], this suggests that no more than one to two whisk cycles were sufficient to judge position. Although the animals in our study often did not show the highly sinusoidal vibrissa motion found in other studies [33,43], perhaps due to the physical restriction of the nose poke, the observed time scale of vibrissa motion here is consistent with these frequencies.

### Neural Algorithms

Earlier studies have suggested the calculation of dorsoventral angle from the identity of the contacting row [41,44] and demonstrated discrimination of distance from contact with multiple vibrissae, even in the absence of active whisking [21]. We focused here on the decoding of angle along the axis of vibrissa motion because of the confound arising from that motion, i.e., the environmental meaning of exteroceptive tactile signals changes as an animal moves its vibrissae. Given that this computation can be accomplished using the information associated with a single moving sensor, we consider the algorithms that might be used to transform a contact signal into a spatial position.

A purely motor strategy could have taken advantage of the fixed stimulus locations in our task design. Because only two positions were possible, an animal might learn to focus its now sparse sensory apparatus on one of these two positions and use the presence or absence of a contact event to detect the ***S***+ (or equivalently, ***S−***) condition without considering both sensory and motor cues. This is a single vibrissa approximation of a labeled-line strategy (Figure 1A), which requires motor control sufficient to hold a lone sensor near an expected target. This approach was ruled out by the motion patterns of the vibrissa; in both animals for which we tracked vibrissa position, the scanning motion was not restricted to the region of a single stimulus (Figure 6G). At the opposite extreme, the wealth of sensory information available from the vibrissa follicle suggests a purely sensory strategy that might arise from differences in the nature of contact as a function of position. The existence of separate on- and off-contact touch signals [28] allows for the possibility that the nervous system determines the duration of a contact and converts it into a spatial position, especially for cases such as sinusoidal motion where different positions result in characteristic contact durations. This temporal delay scheme is contraindicated by the similarity of the measured contact durations (Figure 6I). Finally, a strategy that uses both sensory and motor signals, but with low resolution, would be to position a vibrissa such that it approaches the two stimulus positions from different directions, e.g., during protraction versus retraction. The measured broad heterogeneity of velocity approaching contact (Figure 6H) makes it unlikely that such gross directional cues encoded stimulus position.

The most parsimonious remaining explanation for the computation needed to derive spatial position from a contact event is the integration of touch with kinesthetic information about the location of the vibrissa at the time of contact. Although there is no evidence of proprioceptive muscle spindles in the mystacial pad [45], physiological studies have shown that a reafferent motor signal from the pad is present at levels from the trigeminal ganglion [28,46] to thalamic nuclei [47] to primary somatosensory cortex [26,29,48,49]. The evidence described here argues that this positional information can be used behaviorally as a reference against which to interpret sensory contact, informing spatial perception of objects near the head. Preliminary electrophysiological data show how the fusion of these two signals can occur in the brain [50].

Previous theoretical studies have taken advantage of the rhythmic nature of exploratory whisking to suggest neuronal circuits that might compute spatial location given periodic spike trains representing both contact and vibrissa motion [36,51–53]. The mathematical formulation of this class of algorithm, however, requires multiple rhythmic whisk cycles to establish a phase reference. The animals in our study and another study with head-restrained animals trained to touch objects with their vibrissae [54] do not appear to precede contact with periodic whisking (Figure 6C and 6F) (Figure 3A of Sachdev et al. [54]). This ability to form a spatial percept from oscillatory but irregular vibrissa motion, within one to two cycles after whisking onset, argues against mechanisms that depend on periodicity. A more-direct circuit that performs the same computation would compare spiking in contact-sensitive and position-sensitive cells of varying preferred position. Ongoing physiological studies characterizing these neuronal interactions suggest that this comparison can in fact integrate tactile and haptic streams [50]. Finally, a last constraint on any proposed neuronal implementation of a spatial decoding algorithm should at least account for resolution sufficient to distinguish contacts separated by 15°. Directed studies of the threshold for absolute angular discrimination are required to establish the psychophysical limits of spatial acuity, as has been done for the bilateral comparison of distance [21] and angle [55].

### The Vibrissa System and Sensorimotor Integration

Our behavioral task was designed to isolate a sensory process that is ordinarily used in concert with other behaviors. Earlier studies involving spatial behaviors support the notion that the vibrissae serve as binary detectors in freely exploring animals [20], and this may be the ethologically more typical usage when an animal can orient its head following contact. Indeed, upon detection of the salient and asymmetric stimulus used in this study, some animals in our study reflexively oriented to explore the stimulus further, perhaps bringing their microvibrissae to bear. Although we could not determine whether those animals had judged stimulus position before orienting, we note that refined rostrocaudal information is necessary if a rat is to orient rapidly following contact without further search. Although the current study does not attempt to characterize the role of angular perception during natural activity, we note that the vibrissae are involved in activities with complex spatial demands, such as navigation [14,44], aggression [56], and swimming [57] (for a general review of the classic literature, see Gustafson and Felbain-Keramidas [7]). This ubiquitous role suggests that these sensorimotor organs paint a richer picture of the tactile world than would be possible from binary detection alone. When taken with recent work on the role of the vibrissae in the transduction of fine texture [10–13], the proportionally large neural territory given to vibrissa processing emerges as the potential locus of integration for multiple modalities.

A practical advantage to the study of integration processes in the vibrissa system lies in the large body of work on the plasticity, anatomy, and sensory response properties [36,58–62] related to the vibrissa primary somatosensory or barrel cortex. A variety of recent studies take advantage of this growing body of literature and use the vibrissa system to develop modern experimental methodologies [63–65]. In recognition of the requirements of these techniques, our choice of a go/no-go paradigm, rather than a two-alternative forced choice design [66], was in part motivated by the hope of eventually measuring behavior in animals head-fixed for imaging or intracellular studies [29,63,67–69]. The present work demonstrates that rats can use this popular model system to integrate feed-forward sensory events with motor feedback to inform their model of the external world. The elucidation of the circuitry that performs this computation will bring us a step closer to understanding how sensorimotor loops derive the perception of space from the sensation of touch.

## Materials and Methods

Our initial cohort of behavioral subjects consisted of 24 Long-Evans rats, 14 of which reached the stimulus training stage of the study (Figure 3). All of these animals were females of 100–200-g mass at the start of training. All procedures involving animals conformed to National Institutes of Health guidelines and were approved by the Institutional Animal Care and Use Committee at the University of California at San Diego (La Jolla, California, United States).

### Training apparatus.

Animals were trained and tested in a custom operant arena (Figure 2A). The arena was contained in an enclosure 760 mm × 500 mm × 420 mm (not shown in Figure 2) made of 1/4-in. (0.6-cm) acrylic and padded with skinned polyether foam (1 in. [2.54 cm]; NRC 0.8, McMaster-Carr, 5692T49 [http://www.mcmaster.com]) to dampen external sound and light. The enclosure contained a speaker to deliver sound cues, an auditory white-noise mask, a SecuraCam infrared camera (Swann [http://www.swann.com.au]) for monitoring by the trainer, and a low-flow gas line to ensure breathable air. The area available to the animal consisted of an acrylic vestibule, 200 mm on a side, and a 200-mm long × 57-mm inner diameter tunnel, both placed on an acrylic shelf elevated 60 mm above the floor of the external enclosure. Motion through the tunnel was registered by an 880-nm infrared photodiode (Photonic-Devices, PDI-E802 [http://www.photonicdevicesinc.com])/phototransistor (DigiKey, QSE156-ND [http://www.digikey.com]) pair to signal the presence of a rat. The end of the tunnel was fitted with a U-shaped restraint bar that allowed free movement of the head and paws while preventing escape (Figure 2B).

Behavioral output was measured through a water-sealed lever of 25-g activating force (Cherry E73-series switch, DigiKey, CH566-ND), affixed with a plastic crossbar, and placed at the end of the tunnel (Figure 2B). Animals typically rested on the crossbar and activated the lever by briefly raising their paws; early in training, a small spring was often placed to aid this motion. Fluid rewards were delivered to a sip cup cut into a stainless steel dispenser, placed ∼ 60 mm from the end of the tunnel and designed with inflow and outflow ports to control the timing of fluid delivery. Fluid was delivered in 50-μl aliquots through a miniature direct current solenoid valve (Parker-Hannifin, 004-0008–900 [http://www.parker.com]) with a custom timer circuit (University of California at San Diego Physics Electronics Shop) to gate flow from a reservoir and then removed by a similarly controlled solenoid gating a vacuum line. A bright yellow light-emitting diode (587 nm, 1,900 mcd, Radio Shack 276–351 [http://www.radioshack.com]) was wired in parallel with the reward timer to indicate reward availability and interfere with visual dark-adaptation to any residual light in the chamber, e.g., from the camera illumination lamp. This entire fluid delivery system was flushed with enzymatic detergent (MaxiZyme, Henry Schein, #10–7410 [http://www.hsa.ca]) after each training session to prevent build-up of organic solids. This cleaning step was particularly necessary early in the training when chocolate milk was sometimes used as a liquid reward.

Rat head position was fixed by a 13-mm outer diameter brass nose poke cone, painted black to minimize stray reflections, with an analog reflective infrared sensor (940 nm, DigiKey, QRD1114-ND), whose output was taken to an analog comparator (LM319N, DigiKey, 497-1577–5-ND). The comparator was wired as a Schmitt trigger and referenced to a potentiometer adjusted to control nose poke sensitivity. The nose poke was placed close to the fluid dispenser and fixed relative to the stimulus with an aluminum bracket (not shown in Figure 2; see Figure S4). This system allowed some positional ambiguity, as an animal could roll its snout freely while maintaining fixation. However, translational position was reproducibly constrained (Figures 6B, 6E, and S1).

Digital inputs taken from the lever, tunnel sensor, and nose poke sensor were sampled at 16 Hz by a multifunction digital input/output board (National Instruments, AT-MIO-16DE [http://www.ni.com]) in a personal computer running custom LabVIEW software (National Instruments). The software logged the inputs, implemented training logic, and supplied digital control signals for the reward, vacuum, and stimulus valves.

### Tactile stimuli.

The stimuli for the spatial task were 0.86-mm steel rods translated into and out of the vibrissa field by a custom air-piston and guide assembly (Figure S4). Two paired Lexan guide blocks were aligned on Teflon-coated shafts (15 cm long × 0.6 cm deep, McMaster-Carr, 7875K11). The floor of the bottom guide rested on plastic legs 110 mm above the floor of the tunnel. These legs, the top-guide, and the alignment shafts (Figure S4) were left out of Figure 2A for clarity. A carriage block (Figure S6B) with a captured linear bearing was free to slide on the two rostral shafts, and a mirror-image block traveled along the two caudal shafts. Two stainless-steel air cylinders (spring-return, 5-cm travel, McMaster-Carr, 6498K27) were fixed to the top block (Figure S6A), each with its piston bolted to one of the carriage blocks. Air at 140 kPa was delivered to the pistons through a pair of three-way miniature solenoid valves (Parker-Hannifin, 004-0008–900) that were placed outside of the enclosure to minimize noise. These valves were connected to 12 V direct current supplies via power transistors (Mouser, 610–2N6387 [http://www.mouser.com]) for digital control. Inflowing air was passed through a restriction valve, common to both stimuli, which was adjusted to slow piston descent and minimize vibration during stimulus delivery. Outflowing air was similarly restricted, and silicone bumpers were placed between the carriage and guide blocks to damp vibration from the sudden deceleration at the ends of travel. The resulting stimulus descent and ascent took ∼250 ms and ∼150 ms, respectively, for the full 5-cm travel, including an approximate 30-ms delay in both cases for computer processing, solenoid switching, and the build-up of a sufficient change in air pressure. The white-noise audio mask was played continuously in the box to confound any auditory differences between the stimuli.

Both carriage blocks and the bottom guide block (Figure S5B) were drilled with matching hole patterns on a circle of 25-mm diameter with either 15 ° or 7.5 ° spacing. The nose poke was bolted to the bottom guide block and positioned such that a line connecting the caudal edges of both mystacial pads would approximately coincide with the center of this circle. A backstop was constructed by gluing 18-gauge hypodermic tubing to the back of 90-mm lengths of the 0.86-mm diameter stimulus rods. The backstops rested above the stimulus carriage blocks and allowed the stimuli to travel with the carriage blocks, extending from 10–61 mm below the floor of the bottom guide block. This arrangement allowed the angular location of the stimuli to be rapidly adjusted for each animal and was intrinsically safer for the animals, as the stimuli dropped due to gravity rather than direct piston drive. Our typical configuration fixed potential locations for the stimuli at 3.75° + *n*7.5° (where *n* is an integer between −5 and 5), measured relative to a line through the center of the circle described above and perpendicular to the animal's midline. In all cases, one stimulus was placed at a positive angle and another placed at a negative angle; the angular difference and offset were adjusted for each animal so that both rostral and caudal stimuli fell within the range of vibrissa motion (Figure 6A, 6B, 6D, and 6E).

### Video imaging.

High-speed videography was used to characterize the movement of the vibrissae once discrimination was established. An IEEE-1394 monochrome complimentary metal oxide semiconductor camera (Basler Vision Technologies, 602f [http://www.baslerweb.com]) with infrared sensitivity was mounted under the elevated shelf in the training enclosure and focused on the plane of the nose poke using a television lens (f/0.95, 17 mm, JML Optical Industries, 71932 [http://www.jmloptical.com]) and a 45 ° mirror under the stimuli (Figure 2A). Illumination was provided by an infrared lamp (The LED Light, 850 nm peak [http://www.theledlight.com]), which was modified to double output power, while strobed and filtered at 850 nm to reduce leakage into the rat-visible spectrum. The camera was interfaced to a personal computer running custom LabVIEW software and typically acquired 360 × 300 pixel images at 200 Hz with a 1.2-ms exposure. Images were read into a circular buffer, and 600 images were saved to disk 2.5 s after the start of each behavioral trial, so that 0.5 s of pre-trial video was included in each video sequence.

Video images were analyzed in MatLab (The Mathworks [http://www.mathworks.com]). Gross head motion was extracted from the videos to control for cases in which animals were able to break fixation and reach the stimulus before it could be fully withdrawn. Vibrissa motion was estimated from line-scan data extracted from an image column over multiple time points (Figure 6B and 6E). The median column over time was subtracted from each column to remove stationary objects, and custom segmentation and tracking algorithms (D. N. Hill and S.B. Mehta, unpublished) were applied to estimate vibrissa position at each time point. This line-scan analysis was performed on image columns ten pixels to the left and right of the stimulus to avoid contamination from the motion of the stimulus itself, and vibrissa position in the vicinity of the stimulus was interpolated from a linear fit through these left and right position estimates. Over these short regions, vibrissae were well approximated with a linear fit; quadratic fits from three-point estimates showed negligible contributions from the second-order coefficient. For simplicity, line scans were used throughout rather than arcs, and the position of the vibrissa tracks along the vibrissa length varied slightly. In these tracks, the distance of the line-scan column from the mystacial pad at 30° was approximately 15% greater than the distance at 0°. However, the placement of the stimuli was such that there was no systematic difference in the length at which a vibrissa contacted the rostral and caudal stimuli.

To identify potential contact events, the motion of the stimulus was also extracted from the video (Figure S2). Epochs were identified in which the vibrissa trajectory overlapped the stimulus position and showed a velocity of no more than four pixels per frame (Figure 6H). We used this heuristic because we lacked a bona fide detector for stimulus contact and because image overlap of the vibrissa with the stimulus did not necessarily correspond to physical contact in three dimensions. However, this estimate of vibrissa contact was highly accurate, as verified by manual inspection of the video corresponding to a representative sample of contact events.

### Operant shaping.

**Figure 7:**
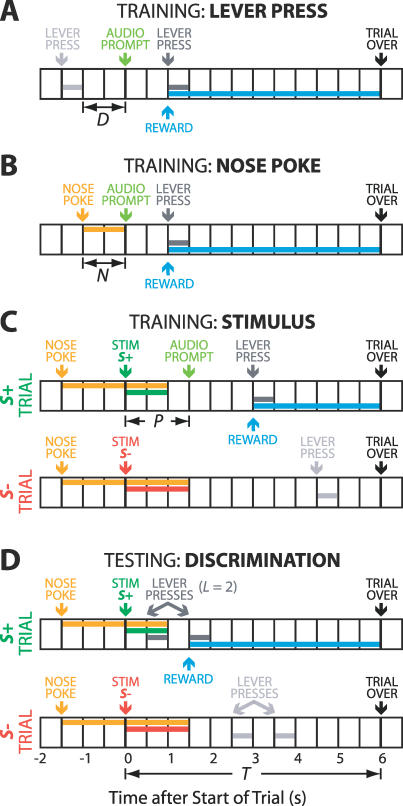
Behavioral Logic for Operant Training and Discrimination Testing All diagrams use 0.5-s intervals here for clarity; the actual sampling rate was 16 Hz. (A) Lever press response training. Animals that waited *D* seconds without emitting a lever press would elicit an audio prompt that signaled the start of a trial. The first lever press response in the following *T* = 6 s was rewarded with a drop of water. At the end of the trial, any remaining water was withdrawn. *D* was increased from 0.25 s to 4 s. (B) Nose poke training. *D* was first increased from 4 to 60 s to decrease the frequency of trial initiated by waiting. Trials could alternatively be initiated if an animal placed its nose in the nose poke for *N* seconds. As this behavior was established, *N* was increased from 0.063 s to 1.5 s. (C) Stimulus training. All trials in this stage were initiated by a 1.5-s nose poke. Each trial was randomly assigned as either ***S***+ or ***S−***, and the start of the trial was signaled by the descent of the corresponding stimulus pin. The pin remained in the vibrissa field until nose fixation was broken or until the trial ended. In ***S***+ trials, the first lever press response after the start of the trial resulted in a reward. If no response occurred within *P* seconds, an audio prompt sounded. *P* was increased from 0.125 s to 6 s. Lever presses were ignored in ***S−*** trials. (D) Stimulus discrimination testing. The audio prompt was eliminated from ***S***+ trials. The number of lever press responses, *L,* required to obtain a reward in ***S***+ trials was increased until a difference between ***S***+ and ***S−*** responses was apparent. This value was typically *L* = 5 or 6; the example here illustrates L = 2. The structure of ***S−*** trials was unchanged.

The discrimination behavior was shaped in three stages through classical operant training techniques (Figure 7) [70]. A small number of parameters were varied in the behavioral program to increment task difficulty, and a large part of training occurred without further experimenter intervention. The parameters used in Figure 7 and in the description below are: *T,* trial length; *D,* delay between trials; *N,* duration of nose fixation; *P,* delay until audio prompt; and *L,* number of lever press responses required to obtain a reward. *P* and *L* were used in rewarded stimulus trials only.

The first stage defined the lever response and brought it under experimental control (Figure 7A). In this stage, animals were required to be in the tunnel without emitting lever press responses for an interval of *D* seconds to elicit an audio prompt, i.e., 300 ms at 2 kHz, which indicated the start of a trial. The first lever press response within *T* seconds following the prompt was rewarded with a 50-μl reward, signaled by a light-emitting diode indicator as described above. At the end of *T* seconds, the trial ended, and any water still in the dispenser was removed by vacuum. This schedule encouraged early responses relative to the audio prompt, because responses that were late but still within *T* seconds after the prompt afforded less time to drink. The trial length *T* was set here to 6 s, and the delay parameter *D* was increased from 0.25 s to 4 s. Human intervention was often necessary in the first several sessions to model the lever press response, but was minimal as *D* increased.

The second stage shaped the nose fixation behavior (Figure 7B). Here, the *D* parameter from stage 1 was increased from 4 s to 60 s, so that the audio prompt was more difficult to elicit by waiting alone. As before, the first lever press response within *T* = 6 s of the prompt was rewarded. A nose poke response of duration *N* seconds short-circuited this delay and immediately caused the start of a trial. The nose fixation duration *N* was increased from 0.063 s to 1.5 s in increments of 0.063 s. Acquisition of the basic nose poke behavior was assisted by increasing the sensitivity of the nose cone sensor and baiting the nose cone with water or chocolate milk; after this acquisition, little intervention was required as the fixation duration parameter *N* was increased.

The third training stage transferred the reward context from the audio prompt to one of the two stimuli in a go/no-go task (Figure 7D). Each animal was randomly and permanently assigned a relative position, either more rostral or more caudal. For each session, the tactile stimulus that occupied this relative angular position was designated ***S***+*,* as opposed to the ***S−*** stimulus at the second position. In this stage, trials could only be initiated by nose fixation. After a nose poke of *N* = 1.5 s, either the ***S***+ or the ***S−*** stimulus, chosen at random with equal probability, was delivered to indicate the start of a trial. For ***S−*** trials, lever press responses in the *T* seconds (*T* = 6 s) following stimulus delivery were unrewarded, and the audio prompt used in earlier stages was never presented. For ***S***+ trials, the first lever press in the *T* seconds (*T* = 6 s) after the start of the trial was rewarded, as before. In ***S***+ trials only, if no lever press response had occurred within *P* seconds after stimulus delivery, an audio prompt was presented to the animal. This delay *P* was increased from 0.125 s to *T* seconds and, as before, any unfinished reward was removed after the trial's end at *T* seconds. Thus, responses to the audio prompt resulted in less time to drink than responses that used the ***S***+ stimulus as a predictor. In both ***S***+ and ***S−*** trials, the stimulus was removed from the vibrissa field if nose poke fixation was broken or the trial ended, whichever came first.

Acquisition of stimulus discrimination was indicated when the number of lever press responses in the first *P* seconds of a trial differed significantly between ***S***+ and ***S−*** trials. The first *P* seconds were used because an animal had access to additional information, in the form of the cue, after *P* seconds. At this point, the audio prompt was eliminated and performance was measured. For some animals, a final parameter was varied during this testing stage. The number of lever press responses *L* (Figure 7D) required to obtain a reward increased from one to five, and the trial length *T* was increased to 8 s to allow time for the longer behavioral response. This manipulation increased the effort necessary to complete a full response in the go/no-go task and discouraged the animal from responding if the stimulus did not predict a reward. The direct use of air puffs as negative reinforcement was unsuccessful at strengthening this asymmetry of response, as it tended to lower the total number trials rather than selectively suppressing ***S−*** responses.

### Fluid restriction and training sessions.

Animals initially acclimated to handling and to chocolate milk (Yoo-Hoo, Cadbury-Schweppes [http://www.cadburyschweppes.com/EN]) from the fluid dispenser in the behavioral chamber over a period of 2–3 wk. Animals were housed in pairs and maintained on a standard light/dark cycle, and both morning and evening training sessions were used. We did not observe any significant effect of time of day or estrous cycle periodicity on behavioral performance. Further, although chocolate milk was used early in training, fluid-deprived animals appeared to perform equally well for water rewards, which were preferred for ease of delivery.

Upon reaching 230 g, animals entered a fluid restriction regimen in which water was removed from their home cage 16–23 h before training to increase motivation. Animals were allowed ad libitum fluid access two days a week, and weights were monitored daily. We used a variable restriction schedule for two reasons. First, the total duration of the training ranged from 6–12 mo, and the long-term health of the animals was a concern. Second, increased pretraining fluid deprivation did not correlate strongly with an increased number of trials per session. Further, the longest deprivation durations we tried, i.e., 24 h, at times caused animals to respond indiscriminately to both ***S***+ and ***S−*** stimuli. Restriction duration was thus tuned separately for each animal.

Daily training sessions were 20–30 min in duration. The physical proximity of the tactile stimulus and response apparatus allowed animals to perform a large number of trials in this amount of time; a total of 50 trials was typical in intermediate stages of training, and a total of 100 trials, of which approximately 50 were rewarded, was typical of well-trained animals on the full discrimination task. These large numbers of trials were needed to obtain statistical evidence of discrimination, and we thus required animals to perform at high rates. As such, five of the initial cohort of 24 rats were removed early in training, i.e., after fewer than 30 sessions, because they performed significantly fewer trials than their littermates. A further five animals were removed at intermediate stages, i.e., after approximately 100 sessions, for performing small numbers of trials after the lever and nose poke were introduced. We did not consider these ten animals in the summary analysis shown in Figure 3, because they were not introduced to the stimulus.

For the remaining 14 animals, the number of sessions required to acquire the lever press response and nose fixation of 1.5 s ranged from 225 for our earliest animals to 61 for animals who started training later in the study. This large reduction in the number of sessions occurred as the training procedures described above were established, and the earlier numbers reflect our adjustments to these procedures rather than the intrinsic time needed to train these behaviors. Further, we chose relatively short session durations to facilitate the concurrent training of multiple animals as we developed the procedures described above. In many cases, however, animals would still be performing trials at the end of a session, albeit at a decreasing pace. It is thus likely that the use of our eventual protocol with longer sessions to obtain more trials per animal per day would have further decreased the training time necessary to achieve this level of behavioral performance.

The number of sessions required to learn the stimulus task was more variable, ranging from 17 to 75, and did not reliably decrease over the course of the study. Further, this measure did not appear correlated with eventual performance as the vibrissae were trimmed.

### Vibrissa trimming.

Animals that successfully discriminated their assigned ***S***+ and ***S−*** stimuli in the absence of the audio prompt (Figure 7D) in multiple consecutive sessions were then trained with only a single vibrissae row intact. At least once per week, animals at this stage were lightly anesthetized on isoflurane for vibrissa trimming. All vibrissae on the left side and all vibrissae on the right side except for the C row were cut. Animals continued training with a single row until they again demonstrated successful discrimination in multiple consecutive sessions, at which point their vibrissae were further trimmed to leave only a single vibrissa intact. This vibrissa was required to be sufficiently long to reach the stimuli and was thus typically chosen to be C1. Animals that performed successfully with a single intact vibrissa were recorded on video as described above and finally challenged with a series of controls.

## Supporting Information

Figure S1 Stimulus Position Relative to the Full Vibrissa Field Video still images of rats in the stimulus assembly nose poke (see also Figure 6B and 6E). (A), (B), and (C) each show both side and bottom views. The two views in each panel were not taken simultaneously but show comparable times in a behavioral trial. The scale bar in all cases is 4 mm.
(A) Rat with all vibrissae intact. Note the large span of the vibrissa field in both rostrocaudal and dorsoventral directions.
(B) Rat with only C row intact. The C3 vibrissa is shown contacting the rostral stimulus. The green arrows point to the location of contact.
(C) Rat with only the C row intact. The γ stradler is shown contacting the caudal stimulus. The red arrows point to the location of contact. This stimulus is 30° caudal to the stimulus shown in (B).
(4.5 MB EPS)

Figure S2 Duration of Stimulus Availability (A) Overlaid stimulus motion traces from 58 ***S***+ and 56 ***S−*** trials. A stimulus descended after an animal had initiated a trial by placing its nose in the nose poke and ascended when the animal first broke nose poke fixation. The descent and ascent motions were estimated from the video recordings and are plotted here. This motion was highly repeatable and smooth; the descending traces appear jagged because the stimuli traversed less than one camera pixel per frame. The discretization evident in the stimulus ascent traces was due to the finite sampling rate, i.e., 16 Hz, of the behavior computer.
(B) Distribution of stimulus availability times. The total time during which the stimulus was in motion was determined from the traces in (A) and is shown histogrammed here for ***S***+ and ***S−*** trials. Note that the animal moves out of the nose poke rapidly, i.e., 200–300 ms, in most ***S***+ trials and waits longer in most ***S−*** trials. This interval is consistent with the time at which the lever press responses first become distinct (Figure 4).
(716 KB EPS)

Figure S3 Evolution of Lever Press Response Distribution The mean cumulative lever press count as a function of time (Figure 4B) does not convey information about the distribution of responses. For example, a mean count of 0.5 responses at 1.0 s could arise from equal numbers of trials with zero responses and with one response, or it could come from many zero response trials and a few trials with two or more responses. Because we relied on the separation of these mean cumulative curves to establish the time scale of the perceptual computation, we show here the complete response distributions corresponding to the mean curves.
(A) Response distributions for 58 ***S***+ trials. At each point in time, the distribution of cumulative lever press counts is plotted such that each column in the image sums to 1.0. The weighted average of each column is equivalent to the mean cumulative response (Figure 4B). The top row shows the fraction of trials that have reached five lever presses as a function of time (equivalent to the cumulative distributions in Figure 4C).
(B) Response distributions for 56 ***S−*** trials. Note that a fraction of the trials never reach five lever press responses. Our conclusion that the ***S***+ and ***S−*** responses diverge after 250 ms (gray arrows in Figure 4B) is reflected here by the overall rightward shift, at all cumulative counts, for the data shown in (B) relative to those shown in (A).
(812 KB EPS)

Figure S4 Complete Stimulus Assembly, Including Support Structures Omitted from Figure 2
The rostral stimulus is shown in a position that would overlap with the vibrissa field, and the caudal stimulus is shown fully retracted.
(A) Three-dimensional perspective. The large horizontal rectangular block is the main platform. Two air-driven pistons are held above this block. Each of these pistons independently drives a carriage that supports a stimulus pin. A hole pattern in the carriages matches the guide pattern inset into the stimulus platform, and these patterns together define repeatable stimulus positions. The Teflon rods on which the carriages travel are shown in brown. The nose poke is the cylindrical black object hanging below the main platform, and it defines the position of the rat relative to the stimulus hole patterns.
(B) Front view.
(C) Side view.
(848 KB EPS)

Figure S5 Detailed Schematics of Stimulus Assembly Main Platform and Guide (A) Main platform. This Lexan platform rested above the rat (as described in Methods and Figure 2) and supported the stimulus assembly.
(B) Stimulus guide. This Lexan block was inset into the main platform and provided guide holes to direct the stimulus pins to various angles on a 25-mm radius. The nose poke was positioned under the main platform such that a line connecting the caudal edges of both mystacial pads would coincide with the center of this circle.
(795 KB EPS)

Figure S6 Detailed Schematics of Stimulus Assembly Piston Support and Carriage (A) Piston support bracket. This fixed Lexan block held the body of the air pistons in place. Precision Teflon rods connecting the bracket to the main platform ensured that piston travel was perpendicular to the stimulus guide.
(B) Stimulus carriages. Each of these blocks was affixed to the shaft of an air piston and allowed to slide along the Teflon rods on a captured linear bearing. Stimulus pins would rest on this carriage and travel with it when the pistons moved.
(786 KB EPS)

## Abbreviations

K-S: Kolmogorov-Smirnov
